# Subarachnoid hemorrhage induces an early and reversible cardiac injury associated with catecholamine release: one-week follow-up study

**DOI:** 10.1186/s13054-014-0558-1

**Published:** 2014-10-30

**Authors:** Reda Salem, Fabrice Vallée, François Dépret, Jacques Callebert, Jean Pierre Saint Maurice, Philippe Marty, Joaquim Matéo, Catherine Madadaki, Emmanuel Houdart, Damien Bresson, Sebastien Froelich, Christian Stapf, Didier Payen, Alexandre Mebazaa

**Affiliations:** AP-HP, Hôpital Lariboisière, Department of Anesthesiology and Critical, Hôpitaux universitaires St-Louis-Lariboisière, University Paris Diderot, U942 INSERM, Paris, F-75475 France; AP-HP, Hôpital Lariboisière, Department of Biology, Hôpitaux universitaires St-Louis-Lariboisière, Paris, F-75475 France; AP-HP, Hôpital Lariboisière, Department of Neuroradiology, Hôpitaux universitaires St-Louis-Lariboisière, Paris, F-75475 France; AP-HP, Hôpital Lariboisière, Department of Neurosurgery, Hôpitaux universitaires St-Louis-Lariboisière, Paris, F-75475 France; DHU Neurovasc Paris Sorbonne, Univ Paris Diderot – Sorbonne Paris Cité, Paris, F-75010 France; AP-HP, Hôpital Lariboisière, Department of Neurology, Hôpitaux universitaires St-Louis-Lariboisière, Paris, cedex 10 F-75475 France; U942 INSERM; University Paris Diderot, Paris, F-75010 France; Department of Anesthesiology and Critical Care, Hôpitaux Universitaires Saint-Louis-Lariboisière, Assistance Publique - Hôpitaux de Paris, Université Paris 7 Denis Diderot, 2 rue Ambroise-Paré, PARIS, Cedex 10 75475 France; CHUM Montréal, Department of Cardiology, Hôpital Universitaire Montréal, 3840, rue Saint-Urbain, Montréal, QC H2W 1T8 Canada

## Abstract

**Introduction:**

The occurrence of cardiac dysfunction is common after subarachnoid hemorrhage (SAH) and was hypothesized to be related to the release of endogenous catecholamines. The aim of this prospective study was to evaluate the relationship between endogenous catecholamine and cardiac dysfunction at the onset and during the first week after SAH.

**Methods:**

Forty consecutive patients admitted for acute SAH without known heart disease were included. Catecholamine plasma concentrations and transthoracic echocardiography (TTE) were documented on admission, on day 1 (D1), and day 7 (D7).

**Results:**

At baseline, 24 patients had a World Federation of Neurosurgical Societies score (WFNS) of one or two; the remaining 16 had a WFNS between three and five. Twenty patients showed signs of cardiac dysfunction on admission, including six with left ventricle (LV) systolodiastolic dysfunction and 14 with pure LV diastolic dysfunction. On admission, norepinephrine, epinephrine, dopamine, B-type Natriuretic Peptide (BNP) and Troponin Ic (cTnI) plasmatic levels were higher in patients with the higher WFNS score and in patients with altered cardiac function (all *P* <0.05). Among patients with cardiac injury, heart function was restored within one week in 13 patients, while seven showed persistent LV diastolic dysfunction (*P* = 0.002). Plasma BNP, cTnI, and catecholamine levels exerted a decrease towards normal values between D1 and D7.

**Conclusion:**

Our findings show that cardiac dysfunction seen early after SAH was associated with both a rapid and sustained endogenous catecholamine release and WFNS score. SAH-induced cardiac dysfunction was regressive over the first week and paralleled the normalization of catecholamine concentration.

## Introduction

Aneurysmal subarachnoid hemorrhage (SAH) is a neurologic emergency. The incidence of the disorder has remained stable over the past 30 years [1] with an aggregate worldwide incidence of about 10.5 per 100,000 person-years [2]. As many as 46% of SAH survivors suffer from long-term cognitive impairment with decreased functional status and quality of life [3–5].

Cardiac dysfunction is frequently observed after acute SAH [6–9]. Meaudre *et al*. [10] found that B-type natriuretic peptide (BNP) and troponin Ic (cTnI) were rapidly and transiently elevated following SAH. Other studies have shown an association between cTnI [9,11], BNP levels [6], clinical SAH severity, and the presence of cardiac dysfunction [11–13]. It has been suggested that the pathophysiology of cardiac injury after SAH is similar to apical ballooning syndrome (Takotsubo or stress cardiomyopathy) that is, in relation to catecholamine endogenous release [14]. Few human studies have shown an association between catecholamine release and cardiac dysfunction at an early stage after SAH, and to our knowledge few human studies have focused on the time course of catecholamine release and cardiac dysfunction during SAH [5,15].

The primary aim of this work was then to confirm the potential association between catecholamine release and cardiac dysfunction during the early phase of SAH. The secondary aim was to study the time course of these relationships over time.

## Methods

### Study design

This prospective observational cohort study was conducted in a university hospital, between June 2008 and May 2009. Our local ethics committee approved the study (CEERB Paris Nord number IRB00006477), and all patients or next of kin provided written informed consent.

Inclusion criteria were as follow: patients >18 years old admitted into the ICU with SAH documented by cerebral computed tomography (CT) scan and angio-CT scan [16]. The neurological status was assessed at the time of ICU admission and graded according to the World Federation of Neurosurgical Societies (WFNS) scale and the CT scan Fisher score.

Exclusion criteria were: patients with known heart disease such as cardiomyopathy, prior myocardial infarction or atrial fibrillation. Patients, families, or referring physicians were interviewed to determine the date and nature of the first signs or symptoms that were clearly attributed to SAH. If delay from first sign or symptom to arrival at our ICU exceeded 48 hours after SAH symptoms, patients were not included.

### Patients

All consecutive patients were admitted to our ICU and were managed according to the recommendations of the French Society for Anesthesia and Intensive Care for SAH [17]. In particular, the culprit cerebral aneurysm identified by angiography was treated as soon as possible by either endovascular coiling or neurosurgical clipping [18]. After the first echocardiography, patients were classified in two groups: patients with no cardiac dysfunction versus patients with either left ventricular systolic (left ventricular ejection fraction (LVEF) <50%) or diastolic cardiac dysfunction defined by the criteria of the American Society of Echocardiography [19]. Patients were also classified in two groups according to the function of the SAH severity: severe SAH (WFNS 3 to 5) or non-severe SAH (WFNS 1 to 2).

### Patient’s management and clinical data collection

Conscious patients were managed with bed rest, hydration, oral nimodipine (Leverkusen) and analgesia. Management of comatose patients included sedation with propofol (New York City, New York) and sufentanyl (Sufenta®, Janssen-Cilag, Belgium), ventilation, enteral feeding, oral nimodipine, and monitoring of intracranial pressure (ICP) in cases of intracranial hypertension.

During the first seven days, each patient received intravenous (iv) isotonic saline at the dose of 30 to 40 ml/kg/day. The presence or absence of cerebral vasospasm by transcranial Doppler was screened daily during the ICU stay. In the event of vasospasm, suspected due to apparition of a new neurological deficit in conscious patients or with asymmetric acceleration of the transcranial Doppler flow, [20] additional fluid infusions were administered. The mean arterial blood pressure was maintained around 100 to 110 mmHg. If the patient remained symptomatic despite fluid loading and high blood pressure, arteriography was performed to treat the vasospasm mechanically (artery dilatation with balloon) or chemically (Nimotop®, Corotrope®) if needed [20].

If clinical signs of intracranial hypertension occurred (ICP >20 mmHg) [21], exogenous norepinephrine was administered to maintain cerebral perfusion pressure above 65 mmHg, and symptomatic hydrocephaly was managed using external ventricular drainage.

Clinical and demographic data including age, sex, body mass index, and body surface area were collected. Exogenous catecholamine administration (for example, norepinephrine) was recorded. Hyponatremia was defined as a sodium level <135 mmol/L for at least two consecutive days [22]. A 12-lead ECG (electrocardiography) was performed daily for seven days. The ECG was considered abnormal if the T-wave was inverted or flattened, the ST segment was elevated or depressed, the QT interval was prolonged, or arrhythmia was present.

### Biomarkers

Blood was drawn in EDTA at baseline, day 1 and day 7, centrifuged within 2 hours and plasma was stored at −80°C until further analysis. After specific sample preparation through solid phase extraction (Chromsystems Instruments®), catecholamine plasma levels (epinephrine, norepinephrine, dopamine,) were analyzed using an isocratic HPLC system and an electrochemical detector (Coularray, ESA). Usual normal values for catecholamine plasma levels were <500, <2500 and <200 pmol/L for epinephrine, norepinephrine and dopamine, respectively [23]. cTnI levels were measured by ELISA (lower limit of detection, 0.01 μg/L). For BNP measurement, arterial blood was withdrawn in EDTA and placed within 30 minutes on a Triage B-Type Natriuretic Peptide test slide and analyzed in the Biosite MeterPlus (Triage®) machine, a point-of-care test based on fluorescence immunoassay. The test has a range of 5 to 5,000 ng/L (normal values <100 ng/L).

### Echocardiography procedure

At the same time as blood analysis, transthoracic echocardiography (TTE) was performed on baseline, day 1, and day 7 using a Vivid-i (General Electrics®) equipped with 2.5-MHz transducers. All TTE was performed by RS (Cardiologist and Intensivist) and FV (Intensivist and anesthesiologist), who are both certified in echocardiography and was performed blinded to all clinical, hemodynamic, and biological data.

Patients were imaged in the supine position. Two-dimensional images were obtained in the standard parasternal and apical views. All echocardiographic data were averaged from three to five end-expiratory cycles. Left ventricular and left atrial dimensions were measured according to the recommendations of the American Society for Echocardiography. LVEF was measured by Simpson's method [24]. Doppler recordings were obtained at a sweep speed of 100 mm/s. Pulsed Doppler was used to record transmitral flow in the apical four-chamber view. Tissue Doppler velocities were acquired at a lateral annular and septal site and E’ mean were calculated as:

$$ \left(\mathrm{E}'\mathrm{mean}=\left(\mathrm{E}'\mathrm{l}\mathrm{a}\mathrm{t} + \mathrm{E}'\mathrm{s}\mathrm{e}\mathrm{p}\right)/2\right). $$

Mitral inflow measurements included early peak (E) and late peak (A) velocities, early peak (E) to late peak (A) ratio and deceleration time (DT) of E velocity. These measurements were analyzed as described previously [25]. The systolic pulmonary artery pressure (SPAP) was estimated using continuous-wave Doppler ultrasound measurement of the peak velocity of a tricuspid regurgitant jet. Tricuspid annular plane systolic excursion (TAPSE) was measured in T-mode at the lateral side on the right ventricle. At baseline, SAH patients were classified in two groups: patients with no cardiac dysfunction versus patients with either LVEF <50% or diastolic cardiac dysfunction defined by criteria of the American Society of Echocardiography [19]. Of note, regional wall motion abnormalities (RWMA) were not recorded in the present study as we were interested to assess global meaningful right and/or left ventricular function abnormalities.

### Statistical analysis

Depending on whether the variables were normally distributed, the *t*-test or Mann-Whitney *U*-test was used to compare quantitative variables and the Chi-square test was used to compare qualitative parameters between patients with or without cardiac dysfunction at ICU admission and according to the clinical severity of SAH. Continuous variables were expressed as median (25th to 75th) percentiles. The analysis of catecholamine, myocardial-injury marker concentration, and TEE-parameter variation over time in each group were analyzed with the Wilcoxon signed-rank test for non-normally distributed variables and the *t*-test for dependent samples in the case of normally distributed parameters. Receiver operator characteristic (ROC) curve analysis was used to determine the best BNP and cTnI cutoff values in order to predict the occurrence of cardiac dysfunction at ICU admission. The level of significance was defined as *P* <0.05. Statistical analysis was performed using Statview© and Medcalc©.

## Results

### SAH patient characteristics

Forty-seven consecutive patients have been admitted to our ICU with non-traumatic SAH. Seven patients were excluded because of known heart disease such as cardiomyopathy (n = 1), prior myocardial infarction (n = 4) or atrial fibrillation (n = 2). The characteristics of the 40 SAH patients included and clinical events are listed in Table 1.

**Table 1:** **Demographic, hemodynamic, echocardiographic, biological and outcome characteristics of SAH patients**

	**All patients**	**WFNS 1 to 2**	**WFNS 3 to 5**	***P*** **-values**
	**n = 40**	**n = 24**	**n = 16**	
**Demographics parameters**				
Age, years	48 (39 to 57)	41.5 (31.5 to 51)	55 (46 to 67)	0.002
Female, n (%)	24 (60)	14 (54)	10 (62)	0.07
Body mass index, kg/m^2^	24.3 (22.6 to 26.1)	24.5 (23 to 26.1)	23.5 (22.3 to 26.2)	0.7
Fisher score	4 (3 to 4)	3 (1.5 to 4)	4 (4 to 4)	0.0006
**Hemodynamic and respiratory parameters on admission**				
Mean arterial pressure, mmHg	85 (80 to 96)	83 (77 to 93)	90 (82 to 107)	0.1
Heart rate, beats per minute	66 (60 to 81)	65 (58 to 76)	76 (62 to 91)	0.04
PaO_2_/FiO_2_ ratio	421 (300 to 452)	445 (407 to 454)	355 (254 to 388)	0.03
PaCO_2_, mmHg	38 (37 to 40)	38 (37 to 40)	40 (35 to 43)	0.51
Mechanical ventilation, n (%)	20 (50)	4 (20)	16 (100)	<0.0001
**Echocardiographic parameters on admission**				
Left ventricular ejection fraction, %	64(44 to 66)	65 (60 to 66)	57 (44 to 66)	0.01
E’, cm/s	9.5 (6 to 13)	12.5 (8.6 to 14.4)	6.5 (6 to 8.7)	<0.0001
TAPSE, mm	22 (19 to 26)	24 (21 to 27)	22 (20 to 23)	0.01
**Myocardial biomarkers on admission**				
B-type natriuretic peptide, ng/L	214 (109 to 374)	202 (79 to 286)	298 (125 to 674)	0.02
Troponin Ic, μg/L	0.026 (0.01 to 1.06)	0.01 (0.01 to 0.28)	0.27 (0.02 to 4.64)	0.006
**Catecholamine levels (see Figure** **1** **)**				
Epinephrine (N: <500 pmol/L)	632 (337 to 1116)	602 (286 to 838)	754 (403 to 1656)	0.04
Norepinephrine (N: <2,500 pmol/Ll)	3,106 (1,913 to 6,803)	2,487 (1,620 to 3,243)	8,451 (4,432 to 35,059)	0.001
Norepinephrine after exclusion of patients receiving exogenous norepinephrine (n = 35, 22 and 13 respectively)	2,754 (1,844 to 4,601)	2,487 (1,620 to 3,243)	5,834 (2,646 to 8,645)	0.006
Dopamine (N: <200 pmol/L)	178 (124 to 347)	155 (121 to 231)	320 (194 to 462)	0.01
Length of stay in ICU, days	10.5 (7.2 to 13.5)	10.4 (6.2 to 12.0)	13.2 (7.5 to 19.7)	0.07
**Complications during ICU stay**				
Vasospasm, n (%)	9 (22)	5 (20)	4 (25)	0.1
Hyponatremia, n (%)	12 (30)	6 (25)	6 (37)	0.4
Seizure, n (%)	9 (22,5)	4 (16)	5 (31)	0.3
Death in ICU, n (%)	7 (17.5)	1 (4)	6 (37)	0.006
**Aneurysm and neurologic management**				
Ventricular derivation, n (%)	13 (32)	2 (8)	11 (68)	<0.0001
Arteriography, n (%)	37 (92)	24 (100)	13 (81)	0.06
Arterioembolization, n (%)	34 (85)	23 (96)	11 (68)	0.02

Data expressed as median (25th to 75th percentiles) or number (%). *P*-values for comparison between World Federation of Neurosurgical Societies (WFNS) scores 1 to 2 and WFNS scores 3 to 5. SAH, aneurysmal subarachnoid hemorrhage; FiO_2_, inspired oxygen fraction; PaO_2_, arterial partial pressure of oxygen; TAPSE, tricuspid annular plane systolic excursion; N, normal value.

The average time between the onset of SAH and ICU admission was 10 ± 9 hours. Thirty-seven patients had arteriography on admission: one or more aneurysms were subsequently diagnosed in 35 patients (34 treated by embolization and one by surgery) and no aneurysms were diagnosed in 2 patients; 3 patients died before arteriography. ICU mortality in the studied patients with SAH was 17.5% (7/40) with much greater mortality in patients with WFNS scores 3 to 5 than in those with WFNS scores 1 to 2 (37 versus 4%; *P* = 0.006).

### Cardiac dysfunction in SAH patients

The average time between ICU admission and the completion of the first echocardiography was 14 ± 10 hours. Half of the included SAH patients (20/40) had echocardiographic abnormalities at ICU admission. The characteristics of SAH patients with and without cardiac dysfunction on admission are shown in Table 2. Table 3 further shows that left ventricular (LV) diastolic dysfunction assessed by the marked fall in the E wave (64 (50 to 94) versus 97 (83 to 102) cm/s, *P* = 0.004), in E’ lateral (7 (6 to 9) versus 15 (17 to 24) cm/s, *P* <0.0001), in E’ mean (7 (6 to 8) versus 13 (11 to 16) cm/s, *P* <0.0001) with no concomitant dilatation of the left atrium (15.6 (12.4 to 18.8) versus 15.9 (12.7 to 18.6) ml/m^2^, *P* = 0.59) was present in all 20 patients with echocardiographic abnormalities compared to patients with no cardiac dysfunction on echocardiography. In addition to LV diastolic dysfunction, 6/20 patients had moderate LV systolic dysfunction (LVEF = 40% (25 to 49%), n = 6). Concerning the right ventricle, TAPSE was lower in SAH with LV abnormalities versus no LV abnormalities, though this was not statistically significant (22 (20 to 24) versus 24 (21 to 27), *P* = 0.07). No echocardiographic signs of Takotsubo were seen.

**Table 2:** **Patients' characteristics as a function of presence of cardiac dysfunction on admission**

	**No cardiac dysfunction on admission**	**Cardiac dysfunction on admission**	***P*** **-values**
	**n = 20**	**n = 20**	
Age, years	40 (31 to 50)	57 (46 to 64)	0.003
Female, n (%)	11 (55)	13 (65)	0.5
Body mass index, kg/m^2^, median	24.5 (22.2 to 26.2)	24.8 (22.6 to 26)	0.6
History of high blood pressure, n (%)	2 (10)	6 (30)	0.23
Diabetes mellitus, n (%)	0 (0)	0 (0)	-
Chronic kidney failure, n (%)	1 (5)	0 (0)	0.9
Tobacco use, n (%)	4 (20)	1 (5)	0.34
Fisher score	3 (2 to 4)	4 (4 to 4)	0.003
WFNS score	1 (1 to 2)	4 (2 to 4)	<0.0001
ECG anomalies, n (%)	2 (10)	3 (15)	0.6
Patients receiving norepinephrine, n (%)	2 (10)	3 (15)	0.6
Mechanical ventilation on admission, n (%)	5 (25)	15 (75)	0.0005
Mechanical ventilation at day 7, n (%)	2 (10)	7 (35)	0.02
Vasospasm, n (%)	4 (20)	5 (25)	0.9
ICU death, n (%)	2 (10)	5 (25)	0.1
Length of stay in ICU, days	9.5 (6.2-11.3)	12.0 (7.5-16.2)	0.2

Data expressed as median (25th to 75th percentiles) or number (%). WFNS: World Federation of Neurosurgical Societies; ECG: electrocardiography.

**Table 3:** **Hemodynamic, echocardiographic parameters and biological markers on admission**

	**No cardiac dysfunction on admission**	**Cardiac dysfunction on admission**	***P*** **-values**
	**n = 20**	**n = 20**	
**Hemodynamics and respiratory parameters during echocardiography**			
Systolic arterial pressure, mmHg	123 (110 to 138)	126 (113 to 149)	0.2
Diastolic arterial pressure, mmHg	63 (58 to 72)	72 (65 to 84)	0.01
Mean arterial pressure, mmHg	81 (76 to 91)	90 (82 to 101)	0.06
Heart rate, beats per minute	63 (57 to 80)	68 (63 to 85)	0.2
PaO_2_/FiO_2_ ratio	445 (421 to 454)	355 (254 to 422)	0.02
**Echographic parameters**			
Left ventricular ejection fraction, %	64 (61 to 70)	60 (47 to 67)	0.04
Stroke volume, ml	81 (71 to 90)	72 (51 to 87)	0.2
Cardiac output, l/minute	5.2 (4.9 to 6.4)	4.6 (3.9 to 5.7)	0.53
Left atrium volume, ml/m^2^	15.6 (12.4 to 18.8)	15.9 (12.7 to 18.6)	0.59
E wave, cm/s	97 (83 to 102)	64 (50 to 94)	0.004
A wave, cm/s	66 (59 to 74)	65 (53 to 91)	0.4
E/A	1.3 (1.2 to 1.6)	1 (0.78 to 1.1)	0.004
E’ lateral, cm/s	15 (14 to 17)	7.5 (6 to 9)	<0.0001
E/E’ lateral ratio	6 (5.1 to 6.8)	9 (7.1 to 10)	0.0003
E’ mean, cm/s	13 (11.5 to 16.5)	7.2 (6 to 8.2)	<0.0001
PAPs, mmHg	21 (16 to 27)	22 (16 to 32)	0.6
TAPSE, mm	24 (21 to 27)	22 (20 to 24)	0.07
S wave tricuspid, cm/s	15 (14 to 17)	17 (15 to 19)	0.16
**Biological markers**			
BNP, ng/L)	178 (70 to 264)	291 (148 to 619)	0.02
Troponin Ic, μg/ml	0.01 (0.01 to 0.05)	0.4 (0.01 to 4.61)	0.01
Epinephrine, pmol/L	481 (256 to 728)	838 (498 to 1,656)	0.04
Norepinephrine, pmol/L	2,404 (1,580 to 3,363)	4,917 (2,490 to 9,046)	0.02
Norepinephrine after exclusion of patients receiving exogenous norepinephrine (n = 35, 18 and 17 respectively)	2,281 (1,519 to 3,264)	3,222 (2,480 to 7,516)	0.01
Dopamine, pmol/L	155 (123 to 231)	282 (169 to 428)	0.03

Data expressed as median (25th to 75th percentile) or number (%). PAPs, pulmonary arterial pressure systolic; TAPSE, tricuspid annular plane systolic excursion; PaO_2_, arterial partial pressure of oxygen; FiO_2_, inspired oxygen fraction; BNP, B-type natriuretic peptide.

Table 3 also shows that patients with echocardiographic abnormalities had two-fold greater plasma BNP, and forty-fold greater cTnI. Of note, ROC analyses showed that a BNP level >298 ng/L on admission was associated with the presence of echocardiographic abnormalities in SAH patients with sensitivity (Se) of 45% (23 to 68) and specificity (Sp) of 85% (62 to 97), and cTnI >0.469 μg/l was associated with echocardiographic abnormalities with Se of 50% (27 to 72) and Sp of 90% (68 to 99). Table 3 further shows that cardiac dysfunction was associated with greater plasma levels of epinephrine, norepinephrine and dopamine in SAH patients. This was also true, for plasma levels of norepinephrine, after exclusion of the five patients who received exogenous norepinephrine: 3,222 (2,480 to 7,516) versus 2,281 (1,519 to 3,264) pmol/L, *P* = 0.01 with or without cardiac dysfunction respectively. Of note, SAH-induced cardiac dysfunction was associated with a worse PaO_2_/FiO_2_ ratio compared to patients with no cardiac dysfunction (355 (254 to 422) versus 445 (421 to 454), *P* = 0.02).

### Relationship between the degree of cardiac dysfunction in SAH and catecholamine levels

**Figure 1:**
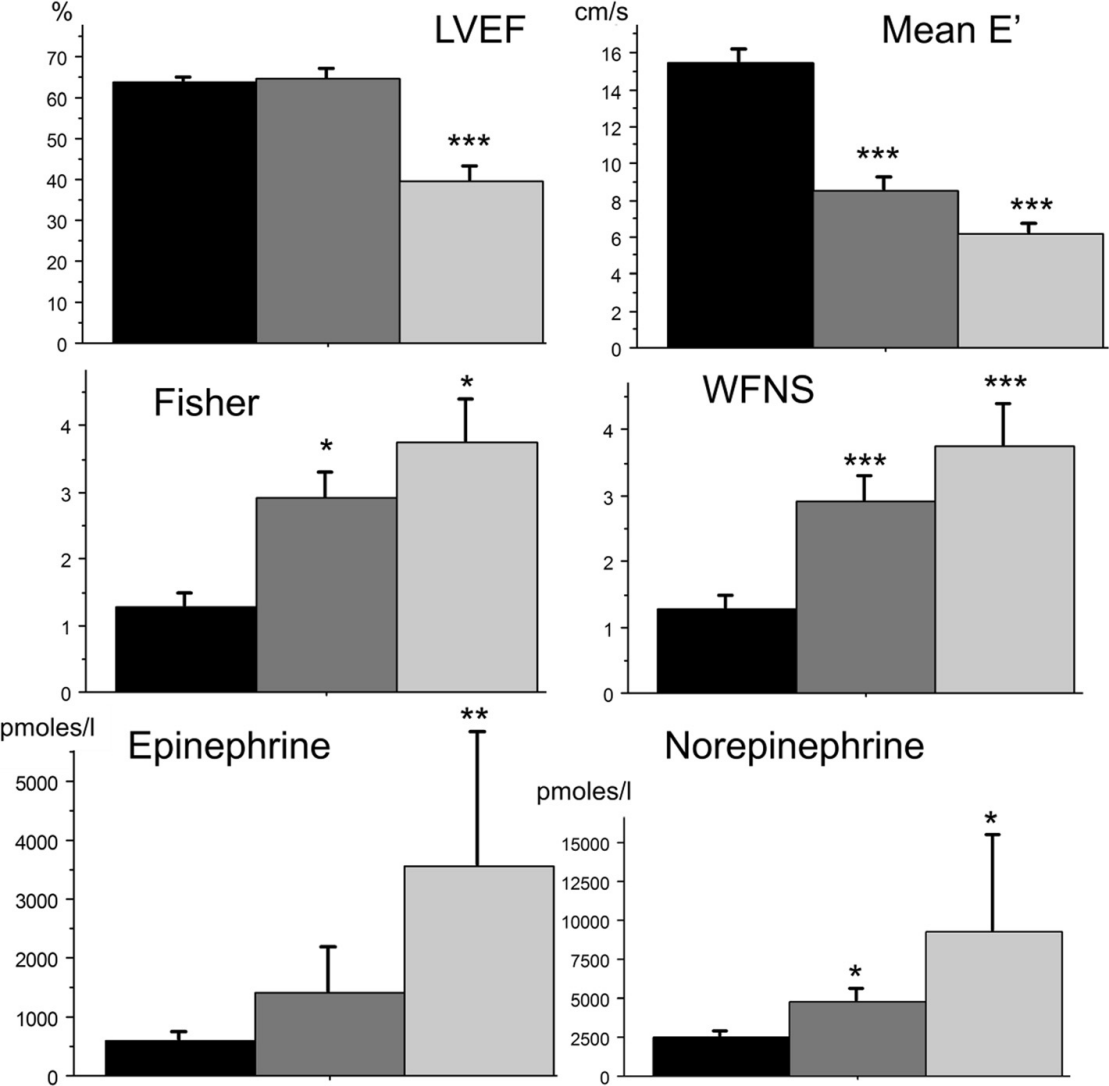
**Clinical, echocardiographic and biological parameters at baseline among patients with systolic-diastolic dysfunction, diastolic dysfunction or no cardiac dysfunction, mean (SD).** Black bars, patients with no cardiac dysfunction (n = 20); dark gray bars, patients with left ventricular diastolic dysfunction (n = 14); light gray bars, patients with left ventricular systolic-diastolic dysfunction (n = 6): *P <0.05, **P <0.01, ***P <0.001 for comparison between cardiac dysfunction and no cardiac dysfunction at baseline. WFNS, World Federation of Neurosurgical Societies; LVEF, left ventricular ejection fraction.

Figure 1 shows that alteration of LV function paralleled catecholamine levels. Indeed, the degree of abnormal echocardiographic findings, namely the combined LV systolic and diastolic dysfunctions (n = 6), was associated with the highest levels of plasma epinephrine and norepinephrine (Figure 1). Furthermore, patients with LV diastolic dysfunction alone had greater plasma levels of epinephrine and norepinephrine than patients with no cardiac dysfunction, though only the association with norepinephrine levels reached statistical significance (*P* <0.05) (Figure 1).

In multivariate analysis (including age, norepinephrine level and WFNS grade), only WFNS grade was statistically associated with cardiac dysfunction on admission (*P* = 0.01). Norepinephrine level was not associated with cardiac dysfunction on admission (*P* = 0.27).

### Time course of cardiac function and plasma catecholamine levels during the ICU stay

**Figure 2:**
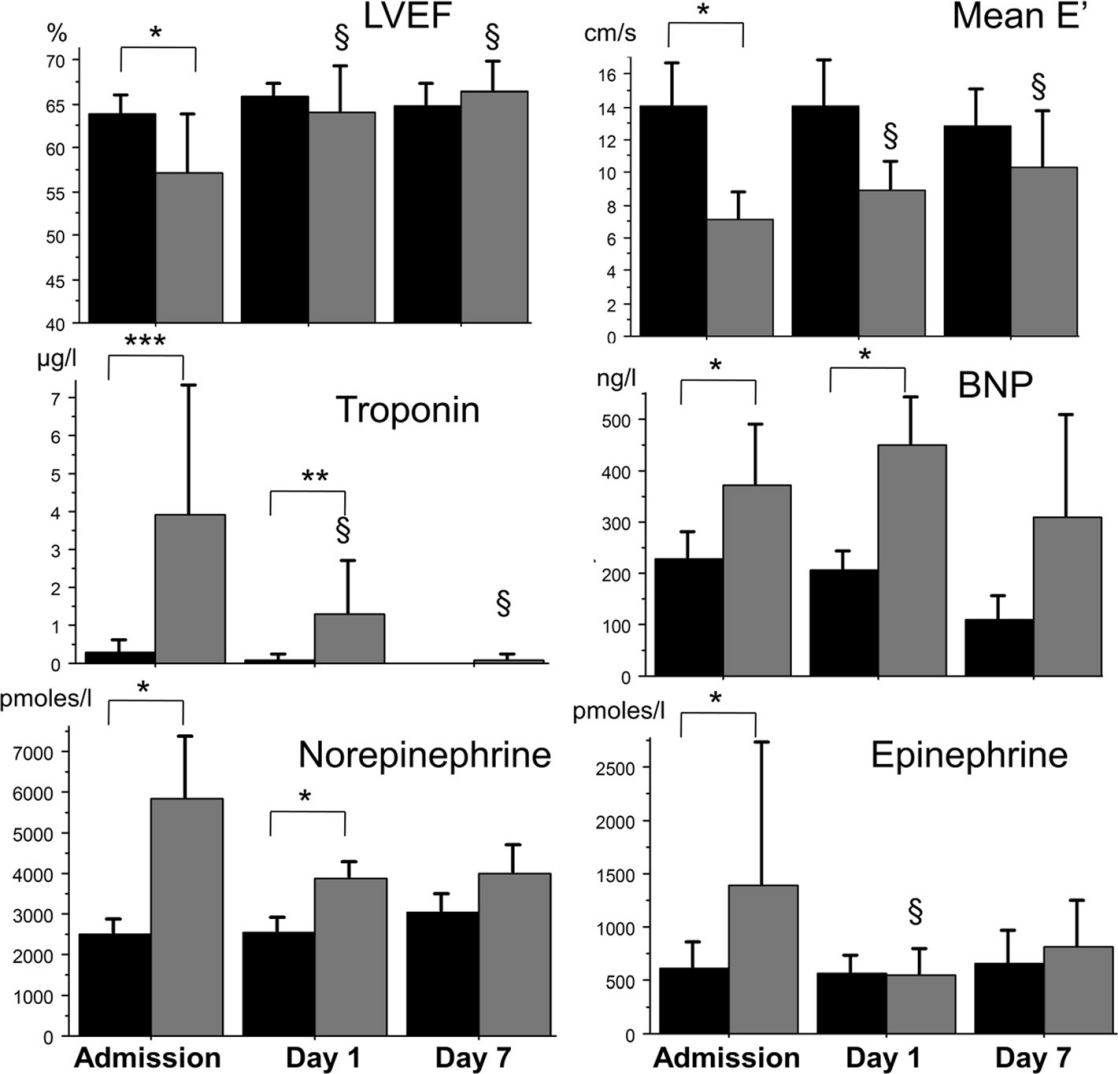
**Time course of echocardiographic signs and biological parameters during the first week after admission in patients with or without cardiac dysfunction at baseline.** Black bars, patients without cardiac dysfunction (n = 20, 19 and 12 at ICU admission, day 1 and day 7); gray bars, patients with cardiac dysfunction (n = 20, 17 and 10 at ICU admission, day 1 and day 7): * *P* <0.05, ** *P* <0.01, *** *P* <0.001 for comparisons between groups; ^§^ *P* <0.05 for comparison with baseline values within the same group. LVEF, left ventricular ejection fraction; BNP, B-type natriuretic peptide.

None of the patients without signs of cardiac dysfunction on admission developed cardiac dysfunction during the following ICU stay. In the group with cardiac dysfunction on admission, a gradual improvement in both LVEF and E’ mean was observed during the first week (Figure 2). Overall, the number of SAH patients with cardiac dysfunction decreased over time: on day 1, 25% of patients (5/20) and on day 7, 70% (14/20) did recover showing a normal echocardiography (defined by LVEF >50% and absence of diastolic dysfunction criteria) [19].

In parallel to improvement in echocardiographic parameters, SAH patients with cardiac dysfunction had undetectable troponin I plasma levels on day 7, while BNP plasma levels decreased but remained above normal values on day 7 (Figure 2). Figure 2 further shows that plasma levels of catecholamines decreased in the group of SAH-patients with cardiac dysfunction from baseline towards levels measured in SAH-patients with no cardiac dysfunction, at day 1 for epinephrine and later for norepinephrine.

### Association with clinical SAH severity

Table 1 show that, the most severe SAH had a global ventricular dysfunction affecting both systolic and diastolic functions. Interestingly, measured cardiac biomarkers and catecholamines were higher in patients with clinical severe SAH compared to non-severe SAH (all *P* <0.05).

## Discussion

Our study confirmed that a large proportion of SAH patients admitted to the ICU showed echocardiographic and biological signs of cardiac injury. As previously described, our study showed that the severity of cardiac injury was associated with the degree of catecholamine release in the plasma of our SAH patients [26] in univariate analysis and with the severity of SAH in multivariate analysis. The evolution of echocardiographic and biological markers paralleled the evolution of plasma levels of norepinephrine and epinephrine and returned in most patients toward normal levels within a week.

### Catecholamine release in SAH patients

SAH-induced myocardial dysfunction is associated with increased blood pressure, increased heart rate and biological and ECG signs of myocardial injury [7,11,12]. Thus, it has long been hypothesized that SAH-induced myocardial dysfunction might be related to release of catecholamines. Catecholamines (epinephrine and norepinephrine) have been shown to be released in the plasma few minutes after the induction of severe SAH in a preclinical dog model [14]. In that preclinical study, catecholamine release lasted less than 2 hours and was followed by a sustained increase in biological markers of cardiac injury and alterations of myocardial function [14]. Our study shows that as reported in the literature [15,27], there is already marked and rapid catecholamine release at admission. The source of SAH-induced catecholamine release remains unknown. It has been suggested that it might be related to hypothalamic injury [28] or the presence of a right temporal hematoma associated with the hemorrhage [27]. This remains to be explored.

In our study, a mild release of catecholamines was associated with an isolated LV diastolic dysfunction, whereas the massive catecholamine release seen in the more severe forms of SAH was associated with the alteration of the global myocardial function including both systolic and diastolic LV functions.

Our study further showed that the restoration of echocardiographic and biological parameters paralleled the decrease in plasma catecholamine release.

### Echocardiographic and cardiac biomarker findings in SAH patients

Our study confirmed that LV diastolic dysfunction was the main echocardiographic finding in SAH patients at ICU admission, as previously described [9,10]. LV diastolic dysfunction was associated with increased troponin I suggesting endocardial ischemia [7,8,10–12,29]. LV diastolic dysfunction was associated with normal left atrium volume, suggesting an acute rather than a chronic alteration in LV diastolic function related to chronic hypertension. Our study extended those findings and showed that the more severe SAH patients exerted the more severe cardiac injury with the highest troponin I and BNP plasma levels and global cardiac dysfunction on echocardiography. Of note, 70% of patients with echocardiographic abnormalities on admission had restored cardiac function at day 7. In parallel, BNP decreased at day 7 though it remained above normal values. Thus, although the majority of SAH patients recovered their heart function by day 7, some SAH patients have persistent echocardiographic or biological abnormalities at day 7 and should be followed longer, as previously described [12].

### Limitations

SAH patients with cardiac dysfunction were older than patients without cardiac dysfunction. However, age difference between patients with and without cardiac dysfunction could not in itself explain SAH-induced LV diastolic dysfunction [19], as this cardiac dysfunction was acute and not chronic, and was regressive during the first week of hospitalization for SAH. Preexisting heart dysfunction might have existed. LV diastolic function assessed using Doppler echocardiography had potential limitations [30]. However, in our study, E, E’ lateral and E’ mean all showed a marked reduction, in favor of a true LV diastolic dysfunction. Furthermore, pulmonary congestion related to LV diastolic dysfunction, was present in patients with those echocardiographic abnormalities.

There was no control group comprising either other non-central nervous system (CNS) injury ICU patients, or patients with any other reason for non-CNS etiology of cardiac dysfunction, in the present study. However, non-severe SAH (WFNS 1 to 2) was used as comparator because the levels of catecholamines in these patients were within the normal range (<2,500 pmoles/L and <500 pmoles/L for epinephrine and norepinephrine, respectively) and they had very few cardiac alterations. Future studies should assess the time course of catecholamines and cardiac function in non-CNS ICU patients. Also, long-term outcome was not recorded in the present study, and the clinical significance of our observation was limited to the acute in-hospital course. Given the single center-design of the study and the few numbers of included-patients, we cannot exclude the possibility of selection bias or other systematic confounders influencing our findings.

As described in the result section, we only found a simple association between cardiac dysfunction and catecholamine levels in univariate analysis. In the multivariate analysis, only WFNS clinical grade predicted cardiac dysfunction but not cathecolamine levels. Thus, the association between catecholamine levels and cardiac dysfunction is not necessarily cause-and-effect and further study should assess the direct role of catecholamines in the occurrence of SAH-related cardiac dysfunction.

## Conclusion

Our study showed that cardiac alterations associated with SAH included LV diastolic function and right ventricular function that both require careful hydration. However, international guidelines recommend maintaining euvolemia from admission in order to prevent vasospasm and subsequent brain injury [31]. In order to prevent pulmonary and other organ congestion related to heart dysfunction, we strongly advise to perform echocardiography on admission and to repeat it until full recovery before starting the triple H therapy. In case echocardiography is not available, our study suggests the possibility to use plasma BNP as a surrogate marker, and to consider BNP plasma level >300 pg/ ml on admission as being strongly associated with cardiac dysfunction. Our study further shows that in one third of SAH patients, cardiac function remained altered as assessed by echo or by high circulating levels of BNP one week after admission for SAH; those patients need prolonged follow up by echo or by measures of circulating BNP.

In summary, the results of the present study show that cardiac dysfunction occurring after SAH was associated with marked, rapid and sustained release of several endogenous catecholamines, and the severity of SAH. Both myocardial alterations and catecholamine levels are regressive during the first week of evolution. Further studies should assess the differential role of catecholamines and brain injury in the occurrence of SAH-related cardiac dysfunction.

## Key messages

- Cardiac dysfunction occurring during SAH is related to catecholamine release
- Cardiac echocardiography is normalized in most patients over the first week
- SAH patients with persistent alteration in cardiac function at day 7 need prolonged cardiac follow up

## Abbreviations

A velocity: late peak velocity
BNP: B-type natriuretic peptide
CNS: central nervous system
CT: computed tomography
cTnI: troponin Ic
DT: deceleration time
E velocity: early peak velocity
ELISA: enzyme-linked immunosorbent assay
FiO_2_: inspired oxygen fraction
HPLC: high performance liquid chromatography
ICP: intracranial pressure
iv: intravenous
LV: left ventricular
LVEF: left ventricular ejection fraction
PaO2: arterial partial pressure of oxygen
ROC: receiver operator characteristic
RWMA: regional wall motion abnormalities
SAH: aneurysmal subarachnoid hemorrhage
Se: sensitivity
Sp: specificity
SPAP: systolic pulmonary artery pressure
TAPSE: tricuspid annular plane systolic excursion
TTE: transthoracic electrocardiography
WFNS: World Federation of Neurosurgical Societies
